# Association between dietary fiber intake and suicidal ideation: a cross-sectional survey

**DOI:** 10.3389/fnut.2024.1465736

**Published:** 2024-10-30

**Authors:** Huaying Huang, Jianjiong Fu, Keyu Lu, Yaming Fu, Pan Zhuge, Yu Yao

**Affiliations:** ^1^Department of Endocrinology and Metabolism, Affiliated Jinhua Hospital, Zhejiang University School of Medicine, Zhejiang, China; ^2^Department of Neurology, Zhuji Affiliated Hospital of Wenzhou Medical University, Zhejiang, China; ^3^Department of Neurology, Affiliated Jinhua Hospital, Zhejiang University School of Medicine, Zhejiang, China; ^4^Department of Otorhinolaryngology Head and Neck Surgery, Affiliated Jinhua Hospital, Zhejiang University School of Medicine, Zhejiang, China

**Keywords:** cross-sectional study, dietary fiber, NHANES, suicidal ideation, nine-item patient health questionnaire

## Abstract

**Background:**

Dietary fiber is beneficial for improving mental health. However, few studies have evaluated the relationship between fiber-rich food and suicidal ideation. Thus, we aimed to assess whether dietary fiber consumption was associated with the risk of suicidal ideation.

**Methods:**

Data of 21,865 American adults were retrieved from the National Health and Nutrition Examination Survey (NHANES). Logistic and restricted cubic spline regression analyses were performed in order to evaluate the association of dietary fiber intake with suicidal ideation, as indicated by item nine of the nine-item Patient Health Questionnaire (PHQ-9). These analyses took into consideration several confounding factors that may potentially influence the results.

**Results:**

Herein, we detected an L-shaped association between dietary fiber intake and the risk of suicidal ideation. For the most conclusive model, an increase of 1 g/1000 kcal/d in dietary fiber intake was accompanied by a 5% reduction in the risk of suicidal ideation. The inflection point of the L-shaped association was located at 7.8 g/1000 kcal/d. When dietary fiber intake exceeded the above level, the risk of suicidal ideation no longer decreased.

**Conclusion:**

Our findings of reduced risk of suicidal ideation in people with higher dietary fiber intake suggest the potential clinical and public health value of dietary fiber. Interventional investigations are warranted to prove whether adhering to a high-fiber diet prevents and reduces suicidality.

## Introduction

More than 700,000 people worldwide commit suicide every year, which brings a heavy psychological and economic burden to families and society (1). A goal to decrease the number of suicide fatalities by 33% in each member state from 2013 to 2030 has been proposed by the World Health Organization (WHO) (2). Based on ideation-to-action theories of suicide, suicidal ideation is the precursor of suicide attempts and in turn causes suicide (3). Approximately 1.4% of psychiatric patients experienced complete suicide in the first year after the expression of suicidal ideation (4). Therefore, identifying modifiable risk factors for suicidal ideation is beneficial for preventing suicide.

Dietary intervention has shown great potential for the prevention and treatment of affective disorders. Numerous indicators have been designed to evaluate the quality of dietary patterns, such as Healthy Eating Index (HEI) (5), Alternative Healthy Eating Index (AHEI) (6), Dietary Approaches To Stop Hypertension Score (DASH) (7), Mediterranean diet (MD) adherence indexes (8), and Dietary Inflammation Index (DII) (9). By means of these indicators, researchers have intuitively exposed the association between healthy dietary patterns and lower risks of anxiety disorder (10, 11), major depression (12), and bipolar disorder (13). The gut microbiota plays an important mediating role in these associations. The differences in dietary structure cause changes in the composition of gut microbiota, which affects the activity of the hypothalamic–pituitary–adrenal axis through the microbiota-gut-brain axis, thereby affecting mental health (14, 15).

Dietary fiber is a group of nondigestible polysaccharides originating from plants (16). Dietary fiber or foods rich in dietary fiber are positive components of the above diet quality indicators. Although dietary fiber cannot be digested and absorbed by the human body, it can regulate the gut microbiota, thereby inhibiting inflammation in the body and affecting neurotransmitters (17). The existing evidence has strongly confirmed that increasing dietary fiber intake improves mental health. A recent meta-analysis showed that an increase of 1 g in dietary fiber intake reduces the risk of depression by 24% (18). By contrast, few studies explored how dietary fiber intake influences suicidal behaviors. Based on the beneficial characteristics of dietary fiber, we speculated that food with high fiber has the possibility of preventing suicidal behaviors. Thus, the goal of this study was to examine the association between dietary fiber intake and suicidal ideation in a representative American population.

## Methods

### Study population

The Centers for Disease Control and Prevention (CDC) conducts the National Health and Nutrition Examination Survey (NHANES) program to evaluate the health and nutritional condition of the American population. In NHANES, the target population was the resident civilian noninstitutionalized population of the United States, which excluded all persons in supervised care or custody in institutional settings, all active-duty military personnel, active-duty family members living overseas, and any other persons residing outside the 50 states and District of Columbia. A complex, multistage, probability sampling design is deployed to select a nationally representative sample of around 5,000 individuals each year. The NHANES utilizes health interviews and physiological examinations to gather participants’ data on demographics, socioeconomics, dietary habits, health-related inquiries, physical examination, and laboratory tests. The NHANES procedures received approval from the Ethics Review Board of the National Center for Health Statistics, and documented consent was obtained from participants.

**Figure 1:**
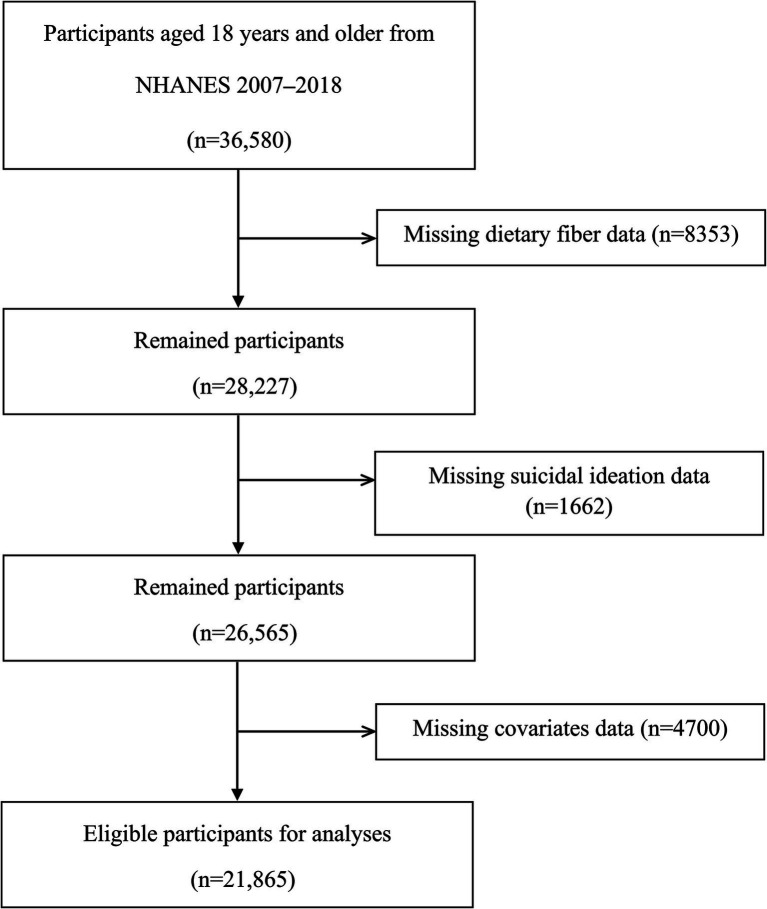
Flowchart of participant selection for this study.

Using the NHANES data, this investigation utilized a cross-sectional design to explore the relationship between dietary fiber intake and suicidal ideation. The original inclusion criteria for this study encompassed all participants from the NHANES 2007–2018 cycles who were 18 years old or older, resulting in a total of 36,580 individuals. After excluding 8,353 participants who did not provide dietary fiber data, 1,662 participants who did not provide suicidal ideation data, and 4,700 participants who did not provide full covariate data, our study covered a total of 21,865 individuals (Figure 1).

### Dietary fiber intake

Data about dietary fiber, as well as other essential nutrients and energy consumption, were acquired by employing two dietary recall interviews. The primary dietary interview was administered in-person in the mobile examination center (MEC), while the subsequent interview was carried out by phone over a period of 3–10 days. A detailed list of foods, beverages, and water consumed during the 24-h period prior to the interview was collected to derive the dietary intake data. Dietary fiber intake was averaged from two dietary interviews. To mitigate the influence of the overall consumption of energy on the results, the dietary fiber intake was corrected for total energy intake using the nutrient density method (19).

### Assessments of suicidal ideation

The nine-item Patient Health Questionnaire (PHQ-9) involves nine questions and is widely used to estimate respondents’ psychological well-being during the last two weeks (20). Suicidal ideation was evaluated by analyzing the answer to item nine of the PHQ-9, which asked: “Over the last two weeks, how often have you felt that you would be better off dead, or hurting yourself in some way?.” Participants with a score of 1–3 were considered to have suicidal ideation. Removing item nine, the first eight questions (PHQ-8) were employed to compute a depression score (21). A cutpoint ≥10 was used to identify depressive symptoms.

### Covariates

Covariates of interest consisting of sociodemographic, lifestyle, health status, and dietary factors were deemed potential confounders. Sociodemographic variables comprised age, sex, race, education level, marital status, and the family income-to-poverty ratio. Lifestyle variables included smoking, drinking, and physical inactivity. Smoking history was characterized by the consumption of at least 100 cigarettes throughout the course of one’s lifetime. Drinking history is the practice of consuming at least 12 alcoholic drinks throughout a span of one year. Physical inactivity was defined as less than 150 min of moderate-intensity aerobic activity per week, less than 75 min of vigorous-intensity aerobic activity peer week, and less than an equivalent combination of moderate- and vigorous-intensity aerobic activity. Health status contained body mass index (BMI), waist circumference, hypertension, diabetes mellitus, hyperlipidemia, coronary heart disease, stroke, cancer, and depression. The diagnostic criteria for the aforementioned diseases are consistent with previous research (22). Dietary factors consisted of carbohydrate, unsaturated fatty acids, sodium, and total energy. Like dietary fiber intake, the intake of these nutrients was averaged from two dietary interviews.

### Statistical analysis

Categorical variables were represented utilizing unweighted frequency and weighted percentage, while continuous data was shown using weighted median and interquartile range. The intergroup variations in continuous and categorical variables were assessed utilizing the Wilcoxon rank–sum test and the chi-squared test with Rao and Scott’s second-order adjustment, respectively. Dietary fiber intake was considered as continuous variables and categorized into four quartiles as categorical variables. Logistic regression analysis was conducted to ascertain the relationship between dietary fiber intake and suicidal ideation. The Crude Model failed to include any potential confounding variables. Model 1 included adjustments for age, sex, and all variables with *p* < 0.1 in the univariate analysis except for depression; Model 2 was further adjusted for depression.

A restricted cubic spline (RCS) regression model was employed to investigate the potential nonlinear relationship between dietary fiber intake and suicidal ideation (23). The model included five knots set at the 5, 27.5, 50, 72.5, and 95th percentiles. The median dietary fiber intake was the reference point. If the relationship was nonlinear, the recursive algorithm was used to detect the inflection point with the highest likelihood. And we employed a two-piecewise logistic regression model on each side of the inflection point to investigate the association between dietary fiber intake and suicidal ideation. The RCS and two-piecewise logistic regression models were adjusted for the same covariates as in Model 2. The statistical analyses were performed employing R version 4.2.1, which was created by the R Foundation for Statistical Computing in Vienna, Austria. The threshold for statistical significance was established at a two-tailed *p*-value of less than 0.05. Sample weights were used to illustrate the complex sampling design of the NHANES.

## Results

Table 1 displays the fundamental features of the participants. Out of the 21,865 participants, 781 individuals (3.6%) were found to have suicidal ideation, whereas 3,601 individuals (16.5%) were diagnosed with depression. The population had a median age of 47.0 years, with females making up 51.4% of the total. The majority of residents, namely 68.5%, were non-Hispanic White. The level of daily dietary fiber intake was measured to be 7.8 (5.8–10.3) g/1000 kcal. In contrast to those without suicidal ideation, subjects with suicidal ideation were more likely to be Hispanics, living alone, and smokers. In addition, those with suicidal ideation had lower education, family income-to-poverty ratio, and dietary unsaturated fatty acid and sodium intake levels. On the contrary, they had higher BMI and waist circumference levels. They were also more likely to have diabetes mellitus, coronary heart disease, stroke, depression, and physical inactivity (all *p* < 0.05). The level of dietary fiber intake was lower in the group with suicidal ideation than that in the group without suicidal ideation (6.6 vs. 7.8 g/1000 kcal/d, *p* < 0.001). Table 2 shows the univariate and multivariable-adjusted ORs of various independent variables for suicidal ideation.

**Table 1:** Participants’ characteristics stratified by the presence of suicidal ideation.

	Overall (*N* = 21,865)	Non-suicidal ideation (*N* = 21,084)	Suicidal ideation (*N* = 781)	*P*-value
Age (years)	47.0 (33.0–60.0)	47.0 (33.0–60.0)	48.0 (33.0–59.0)	0.89
Sex, %				0.53
Female	11,247 (51.4)	10,823 (51.3)	424 (53.2)	
Male	10,618 (48.6)	10,261 (48.7)	357 (46.8)	
Race, %				<0.001
Mexican American	3,067 (8.2)	2,942 (8.2)	125 (9.9)	
Other Hispanic	2,126 (5.4)	1999 (5.2)	127 (10.6)	
Non-Hispanic White	9,829 (68.5)	9,497 (68.7)	332 (61.9)	
Non-Hispanic Black	4,593 (10.5)	4,460 (10.5)	133 (10.1)	
Other Race	2,250 (7.4)	2,186 (7.4)	64 (7.6)	
Education level, %				<0.001
Less than high school	4,621 (13.6)	4,345 (13.2)	276 (24.5)	
High school	5,019 (22.7)	4,838 (22.6)	181 (25.2)	
More than high school	12,225 (63.7)	11,901 (64.1)	324 (50.3)	
Marital status, %				<0.001
Married/living with partner	13,227 (63.6)	12,872 (64.1)	355 (48.9)	
Widowed/divorced/separated	4,745 (17.8)	4,494 (17.5)	251 (27.4)	
Never married	3,893 (18.6)	3,718 (18.4)	175 (23.7)	
Family income-to-poverty ratio, %	3.1 (1.5–5.0)	3.1 (1.6–5.0)	1.7 (0.9–3.1)	<0.001
Body mass index (kg/m^2^)	28.0 (24.3–32.7)	28.0 (24.3–32.7)	29.1 (24.7–34.1)	0.017
Waist circumference (cm)	98.2 (87.6–109.7)	98.1 (87.5–109.6)	101.9 (90.6–112.0)	0.005
Smoking, %	9,839 (44.2)	9,374 (43.7)	465 (57.8)	<0.001
Drinking, %	15,153 (74.1)	14,605 (74.1)	548 (73.6)	0.84
Physical inactivity, %	8,453 (33.7)	8,071 (33.4)	382 (42.8)	0.001
Hypertension, %	9,212 (36.3)	8,845 (36.1)	367 (42.1)	0.053
Diabetes mellitus, %	3,705 (12.3)	3,526 (12.2)	179 (15.8)	0.035
Hyperlipidemia, %	12,746 (57.8)	12,242 (57.7)	504 (62.5)	0.092
Coronary heart disease, %	910 (3.5)	856 (3.4)	54 (5.6)	0.023
Stroke, %	788 (2.7)	723 (2.5)	65 (6.7)	<0.001
Cancer, %	2,231 (10.4)	2,133 (10.4)	98 (11.3)	0.49
Depression, %	3,601 (17.5)	3,071 (15.7)	530 (68.3)	<0.001
Energy (kcal/d)	1978.5 (1541.5–2532.6)	1980.0 (1548.5–2531.5)	1891.6 (1414.6–2559.7)	0.090
Dietary carbohydrate (g/100 kcal/d)	12.0 (10.5–13.5)	12.0 (10.5–13.5)	12.3 (10.4–13.9)	0.073
Dietary unsaturated fatty acids (g/100 kcal/d)	2.2 (1.9–2.6)	2.2 (1.9–2.6)	2.1 (1.7–2.5)	<0.001
Dietary sodium (mg/100 kcal/d)	165.0 (141.2–192.3)	165.2 (141.5–192.5)	160.4 (132.5–185.2)	0.005
Dietary fiber (g/1000 kcal/d)	7.8 (5.8–10.3)	7.8 (5.8–10.4)	6.6 (4.9–9.3)	<0.001
Quartiles of dietary fiber				<0.001
Q1: ≤ 5.8 g/1000 kcal/d	5,466 (25.2)	5,203 (24.7)	263 (38.1)	
Q2: 5.9–7.8 g/1000 kcal/d	5,467 (25.4)	5,271 (25.4)	196 (25.1)	
Q3: 7.9–10.5 g/1000 kcal/d	5,465 (25.9)	5,298 (26.1)	167 (20.1)	
Q4: ≥10.6 g/1000 kcal/d	5,467 (23.5)	5,312 (23.7)	155 (16.8)	

**Table 2:** Univariate and multivariable logistic regression analyses to identify the association between various independent variables and suicidal ideation.

Variables	Univariate model		Multivariable model	
	OR (95% CI)	*P*-value	OR (95% CI)	*P*-value
Age (years)	1.00 (0.99–1.01)	0.78	1.00 (0.99–1.01)	0.61
Sex				
Female	Ref		Ref	
Male	0.93 (0.73–1.18)	0.53	1.34 (0.99–1.81)	0.059
Race				
Mexican American	Ref		Ref	
Other Hispanic	1.69 (1.15–2.48)	0.008	1.48 (0.97–2.27)	0.068
Non-Hispanic White	0.75 (0.55–1.01)	0.061	0.70 (0.49–1.00)	0.047
Non-Hispanic Black	0.79 (0.56–1.11)	0.18	0.65 (0.43–0.98)	0.039
Other Race	0.85 (0.53–1.36)	0.49	0.92 (0.50–1.70)	0.80
Education level				
Less than high school	Ref		Ref	
High school	0.60 (0.44–0.83)	0.002	0.74 (0.52–1.05)	0.10
More than high school	0.42 (0.32–0.56)	<0.001	0.76 (0.55–1.05)	0.10
Marital status				
Married/living with partner	Ref		Ref	
Widowed/divorced/separated	2.05 (1.57–2.68)	<0.001	1.26 (0.94–1.68)	0.11
Never married	1.68 (1.25–2.26)	<0.001	1.37 (0.95–1.97)	0.093
Family income-to-poverty ratio	0.70 (0.64–0.76)	<0.001	0.81 (0.72–0.90)	<0.001
Body mass index (kg/m^2^)	1.02 (1.00–1.03)	0.013	0.99 (0.95–1.03)	0.50
Waist circumference (cm)	1.01 (1.00–1.01)	0.006	1.01 (0.99–1.02)	0.56
Smoking	1.76 (1.39–2.23)	<0.001	(0.80–1.39)	0.71
Drinking	0.97 (0.76–1.25)	0.84	NA	
Physical inactivity	1.49 (1.17–1.89)	0.001	1.10 (0.81–1.50)	0.53
Hypertension	1.29 (1.00–1.66)	0.054	0.92 (0.67–1.27)	0.62
Diabetes mellitus	1.35 (1.02–1.80)	0.036	1.00 (0.73–1.37)	>0.99
Hyperlipidemia	1.23 (0.97–1.56)	0.093	1.13 (0.89–1.44)	0.31
Coronary heart disease	1.69 (1.07–2.68)	0.025	1.14 (0.68–1.91)	0.60
Stroke	2.79 (1.96–3.97)	<0.001	1.66 (1.10–2.49)	0.016
Cancer	1.10 (0.84–1.44)	0.49	NA	
Depression	11.6 (9.11–14.70)	<0.001	11.0 (8.38–14.60)	<0.001
Energy (kcal/d)	1.00 (1.00–1.00)	0.37	1.00 (1.00–1.00)	0.83
Dietary carbohydrate (g/100 kcal/d)	1.06 (1.00–1.11)	0.033	0.97 (0.91–1.04)	0.40
Dietary unsaturated fatty acids (g/100 kcal/d)	0.71 (0.57–0.89)	0.003	0.82 (0.62–1.09)	0.17
Dietary Sodium (mg/100 kcal/d)	1.00 (0.99–1.00)	0.002	1.00 (1.00–1.00)	0.24
Quartiles of dietary fiber				
Quartile 1 (≤ 5.8 g/1000 kcal/d)	Ref		Ref	
Quartile 2 (5.9–7.8 g/1000 kcal/d)	0.64 (0.49–0.84)	0.002	0.71 (0.53–0.97)	0.029
Quartile 3 (7.9–10.5 g/1000 kcal/d)	0.50 (0.36–0.69)	<0.001	0.61 (0.42–0.90)	0.013
Quartile 4 (≥10.6 g/1000 kcal/d)	0.46 (0.34–0.63)	<0.001	0.66 (0.43–1.02)	0.058

CI, confidence interval; OR, odds ratio.

The logistic regression analysis findings for the association between dietary fiber intake and suicidal ideation are shown in Table 3. An inverse association between dietary fiber intake and suicidal ideation was observed in the initial unadjusted model and subsequent adjusted models. With all adjustments implemented on Model 2, it was observed a 5% drop in the likelihood of suicidal ideation for each 1 g/1000 kcal/d increase in dietary fiber intake (odds ratio [OR] 0.95, 95% confidence interval [CI]: 0.91–1.00; *p* = 0.046). To further explore this relationship, dietary fiber intake was classified into discrete intervals (quartiles) for analysis. In the fully adjusted model (Model 2), quartiles 2, 3, and 4 exhibited a 29, 39, and 34% lower probability of suicidal ideation compared to the lowest quartile (quartile 1), respectively.

**Table 3:** Logistic regression analysis to identify the association between dietary fiber intake and suicidal ideation.

	Crude model		Model 1		Model 2	
	OR (95% CI)	*P*–value	OR (95% CI)	*P*–value	OR (95% CI)	*P*–value
Continuous variable	0.91 (0.87–0.95)	<0.001	0.93 (0.89–0.98)	0.004	0.95 (0.91–1.00)	0.046
Categorical variable						
Quartile 1	ref		ref		ref	
Quartile 2	0.64 (0.49–0.84)	0.002	0.71 (0.54–0.95)	0.022	0.71 (0.53–0.97)	0.029
Quartile 3	0.50 (0.36–0.69)	<0.001	0.60 (0.42–0.87)	0.008	0.61 (0.42–0.90)	0.013
Quartile 4	0.46 (0.34–0.63)	<0.001	0.56 (0.37–0.85)	0.007	0.66 (0.43–1.02)	0.058
*P* for trend	<0.001		0.006		0.050	

Model 1: adjusted for age, sex, race, education level, marital status, family income-to-poverty ratio, body mass index, waist circumference, smoking, physical inactivity, hypertension, diabetes mellitus, hyperlipidemia, coronary heart disease, stroke, energy, dietary carbohydrate, dietary unsaturated fatty acids, and dietary sodium. Model 2: adjusted for variables in Model 1 plus depression, CI, confidence interval; OR, odds ratio.

**Figure 2:**
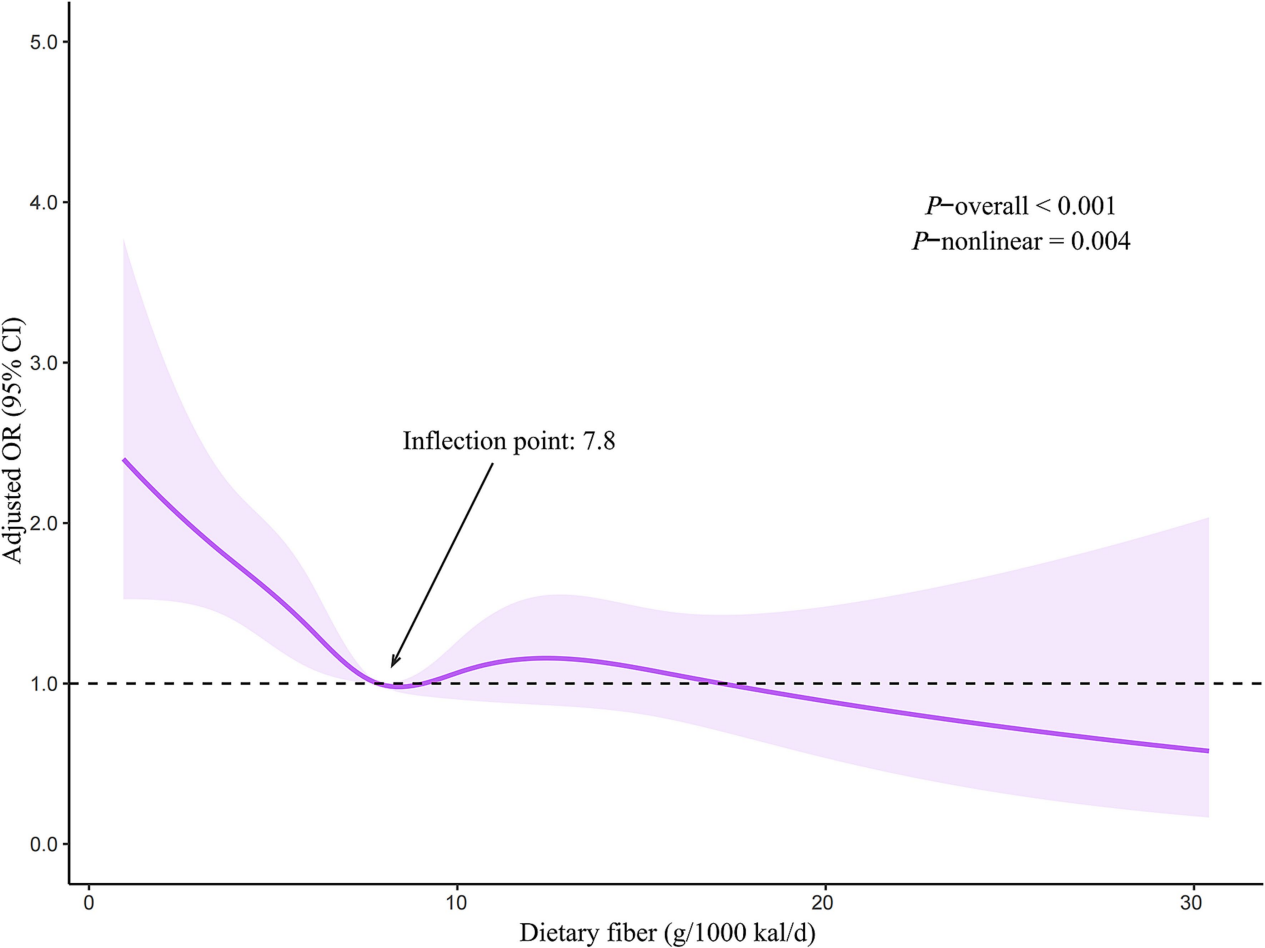
Restricted cubic spline regression model of the association between dietary fiber intake and the risk of suicidal ideation. CI, confidence interval; OR, odds ratio.

The restricted cubic spline regression model demonstrated that dietary fiber intake level was shown to have an inverse relationship with the probability of experiencing suicidal ideation, following a nonlinear pattern (*p*-nonlinear = 0.004) (Figure 2). The inflection point for the L-shaped relationship was identified as 7.8 g/1000 kcal/d. When dietary fiber intake level was < 7.8, it suggested a significant negative association with the risk of suicidal ideation (OR 0.94, 95% CI: 0.89–0.99; *p* = 0.028). And when dietary fiber intake level was ≥7.8, the risk of suicidal ideation was not significantly associated with changes in dietary fiber intake level (Table 4).

**Table 4:** The two-piecewise logistic regression model to identify the inflection point value.

	OR (95% CI)	*P*-value
Fitting by the weighted logistic regression model	0.95 (0.91–1.00)	0.046
Fitting by the two-piecewise logistic regression model		
Inflection point	7.8 g/1000 kcal/d	
Dietary fiber intake <7.8 g/1000 kcal/d	0.94 (0.89–0.99)	0.028
Dietary fiber intake ≥7.8 g/1000 kcal/d	1.01 (0.98–1.04)	0.64
*P* for log-likelihood ratio test	0.064	

Adjusted for variables in Model 2. CI, confidence interval; OR, odds ratio.

## Discussion

By using the data of the representative American population, we observed lower levels of dietary fiber intake in persons who had suicidal ideation compared to those who did not. The association between dietary fiber intake and suicidal ideation was independent of sociodemographic, lifestyle, health status, and dietary factors. There is an L-shaped relationship between levels of dietary fiber consumption and suicidal ideation risk, and the threshold was 7.8 g/1000 kcal/d.

The term suicidal behaviors encompass any suicidal thought or actions, that is suicidal ideation, suicidal attempt, and suicide (24). The previous evidence regarding the link between dietary fiber and suicidal ideation is very scarce. The only finding is that dietary fiber intake is associated with suicide attempts. A cross-sectional study involving 7,631 young adults showed significantly lower dietary fiber intake in suicide attempters compared to non-attempts (25). Our investigation is the first to explore the association between dietary fiber intake and suicidal ideation. The analysis results provide new evidence for the possible improvement of suicidal behavior by elevating dietary fiber intake. This result has significant implications for medical practice and public health. For medical staff, especially nurses, when providing comprehensive care for patients with low dietary fiber intake, they should strengthen safety inspections and education for this group of people to avoid the occurrence of potential suicide incidents. Further researches should explore how high dietary fiber intake modifies the risk of suicidal ideation in the future, in order to establish a causal relationship.

A high-fiber diet is characterized by high consumption of fiber-rich fruits, vegetables, and whole-grain foods (26). In 221,081 Korean adults from the Korea Community Health Survey (KCHS) database, the consumption of fruits and vegetables less than two times per day increased the likelihood of suicidal ideation by 15% (27). Another investigation using the Third National Health and Nutrition Examination Survey (NHANES III) data showed low vegetable consumption in men suicidal attempters and insufficient fruit consumption in women suicidal attempters (28). Healthy Eating Index (HEI) including fruit and vegetable intake has also been found to be inversely correlated with the risk of suicidal ideation (29). Collectively, these findings supported our results that dietary fiber intake was inversely associated with the risk of suicidal ideation. Consuming high-fiber foods to increase dietary fiber intake has the potential to reduce the risk of suicidal ideation.

The mechanism by which dietary fiber affects suicidal ideation remains unclear. Given that dietary fiber is indigestible, its protective effect is likely mediated by gut microbiota. The fermentation of dietary fiber by microbiota produces short-chain fatty acids (SCFAs), which affect metabolism and have anti-inflammatory action (30). In turn, dietary fiber improves the structure and diversity of gut microbiota (30). We have proposed some possible biological explanations for the association between dietary fiber and suicidal ideation. First, dietary fiber can regulate the microbial composition of the gut microbiota to increase serotonin synthesis (31). Serotonin is an important neurotransmitter involved in emotional regulation, and its low levels are associated with an elevated risk of suicidal behaviors (32). Second, fermentation products SCFAs can inhibit histone deacetylases (33) and thereby modify epigenetics and upregulate the levels of brain-derived neurotrophic factor (34), resulting in decreased risk of suicide. Third, SCFAs exert anti-inflammation by reducing intestinal membrane permeability and pH, as well as activating G-protein–coupled receptors (17). The increase of inflammation levels reflected in C-reactive protein (35) and interleukin-6 levels (36) disrupts neurocircuits of specific brain regions associated with suicidality.

The threshold effect of dietary fiber in preventing suicidal ideation was also found in our study. This may be explained by the composition of the gut microbiota. In intervention trials, the effect of dietary fiber supplements on the changes in gut microbiota structure is dose-dependent and has cutoff thresholds (30). According to our results, ensuring that dietary fiber intake is not less than 7.8 g/1000 kcal/d can minimize the likelihood of having suicidal ideation.

A few limitations should be acknowledged before interpreting the results. First, this study is cross-sectional, preventing a causal relationship from being established. The association between dietary fiber consumption and suicidal ideation is probably bidirectional. People with suicidality may tend to adhere to high-carbohydrate and high-fat diets, but lack fruit and vegetable intake. Second, although the item nine of PHQ-9 has been previously used to define suicide ideation, it includes non-suicidal harm, which may affect the results of this research. Third, this study did not distinguish the types (soluble and insoluble) and sources (grains, fruits, vegetables, and legumes) of dietary fiber. The impact on psychological well-being varies depending on the types and sources of dietary fiber (37). Fourth, there may still be several remaining confounders that cannot be totally eliminated, such as other components of a healthy diet and coexisting healthy lifestyle.

## Conclusion

In this study, we discovered an L-shaped association between dietary fiber intake and the risk of suicidal ideation in American adults. The cutoff threshold of dietary fiber intake was 7.8 g/1000 kcal/d. The findings have important implications for both medical practice and public health. Because diet is a modifiable lifestyle, further prospective investigations and randomized controlled trials are required to investigate the effectiveness and safety of fiber-rich food or fiber supplements in protecting against suicidal ideation.

## Data Availability

The raw data supporting the conclusions of this article will be made available by the authors, without undue reservation.

## References

[1] World Health Organization, (2023). Suicide. Available at: https://www.who.int/news-room/fact-sheets/detail/suicide#:~:text=Close%20to%20800%20000%20people,who%20attempt%20suicide%20every%20year.&text=Suicide%20is%20the%20third%20leading,%2D%20and%20middle%2Dincome%20countries (accessed July 1, 2024).

[2] World Health Organization, (2021). Comprehensive Mental Health Action Plan 2013–2030. Available at: https://www.who.int/publications/i/item/9789240031029 (accessed July 1, 2024).

[3] Klonsky ED Saffer BY Bryan CJ. Ideation-to-action theories of suicide: a conceptual and empirical update. Curr Opin Psychol. (2018) 22:38–43. doi: 10.1016/j.copsyc.2017.07.020, PMID: 30122276

[4] Hubers AAM Moaddine S Peersmann SHM Stijnen T van Duijn E van der Mast RC. Suicidal ideation and subsequent completed suicide in both psychiatric and non-psychiatric populations: a meta-analysis. Epidemiol Psychiatr Sci. (2018) 27:186–98. doi: 10.1017/S2045796016001049, PMID: 27989254 PMC6998965

[5] Krebs-Smith SM Pannucci TE Subar AF Kirkpatrick SI Lerman JL Tooze JA. Update of the healthy eating index: HEI-2015. J Acad Nutr Diet. (2018) 118:1591–602. doi: 10.1016/j.jand.2018.05.021, PMID: 30146071 PMC6719291

[6] Chiuve SE Fung TT Rimm EB Hu FB McCullough ML Wang ML. Alternative dietary indices both strongly predict risk of chronic disease. J Nutr. (2012) 142:1009–18. doi: 10.3945/jn.111.157222, PMID: 22513989 PMC3738221

[7] Mellen PB Gao SK Vitolins MZ Goff DC. Deteriorating dietary habits among adults with hypertension. Arch Intern Med. (2008) 168:308–14. doi: 10.1001/archinternmed.2007.119, PMID: 18268173

[8] Bach-Faig A Geleva D Carrasco JL Ribas-Barba L Serra-Majem L. Evaluating associations between Mediterranean diet adherence indexes and biomarkers of diet and disease. Public Health Nutr. (2006) 9:1110–7. doi: 10.1017/S1368980007668499 17378949

[9] Shivappa N Steck SE Hurley TG Hussey JR Hébert JR. Designing and developing a literature-derived, population-based dietary inflammatory index. Public Health Nutr. (2014) 17:1689–96. doi: 10.1017/S1368980013002115, PMID: 23941862 PMC3925198

[10] Richard A Rohrmann S Pestoni G Strippoli MPF Lasserre A Marques-Vidal P. Associations between anxiety disorders and diet quality in a Swiss cohort study. Compr Psychiatry. (2022) 118:152344. doi: 10.1016/j.comppsych.2022.152344 35985108

[11] Sun QZ Wang H Zhang HC Zhang F. Association between the healthy eating index and anxiety among Chinese elderly: a population-based cross-sectional study. Prev Med Rep. (2024) 37:102576. doi: 10.1016/j.pmedr.2023.102576, PMID: 38268617 PMC10805664

[12] Lassale C Batty GD Baghdadli A Jacka F Sánchez-Villegas A Kivimäki M. Healthy dietary indices and risk of depressive outcomes: a systematic review and meta-analysis of observational studies. Mol Psychiatry. (2019) 24:965–86. doi: 10.1038/s41380-018-0237-8, PMID: 30254236 PMC6755986

[13] Lojko D Stelmach-Mardas M Suwalska A. Diet quality and eating patterns in euthymic bipolar patients. Eur Rev Med Pharmacol Sci. (2019) 23:1221–38. doi: 10.26355/eurrev_201902_17016, PMID: 30779092

[14] Lucidi L Pettorruso M Vellante F Di Carlo F Ceci F Santovito MC. Gut microbiota and bipolar disorder: an overview on a novel biomarker for diagnosis and treatment. Int J Mol Sci. (2021) 22:3723. doi: 10.3390/ijms22073723, PMID: 33918462 PMC8038247

[15] Marano G Mazza M Lisci FM Ciliberto M Traversi G Kotzalidis GD. The microbiota-gut-brain Axis: Psychoneuroimmunological insights. Nutrients. (2023) 15:1496. doi: 10.3390/nu15061496, PMID: 36986226 PMC10059722

[16] Anderson JW Baird P Davis RH Ferreri S Knudtson M Koraym A. Health benefits of dietary fiber. Nutr Rev. (2009) 67:188–205. doi: 10.1111/j.1753-4887.2009.00189.x 19335713

[17] Swann OG Kilpatrick M Breslin M Oddy WH. Dietary fiber and its associations with depression and inflammation. Nutr Rev. (2020) 78:394–411. doi: 10.1093/nutrit/nuz072, PMID: 31750916

[18] Fatahi S Matin SS Sohouli MH Gaman MA Raee P Olang B. Association of dietary fiber and depression symptom: a systematic review and meta-analysis of observational studies. Complement Ther Med. (2021) 56:102621. doi: 10.1016/j.ctim.2020.102621, PMID: 33220451

[19] Willett WC Howe GR Kushi LH. Adjustment for total energy intake in epidemiologic studies. Am J Clin Nutr. (1997) 65:1220S–8S. doi: 10.1093/ajcn/65.4.1220S 9094926

[20] Kroenke K Spitzer RL Williams JBW. The PHQ-9—validity of a brief depression severity measure. J Gen Intern Med. (2001) 16:606–13. doi: 10.1046/j.1525-1497.2001.016009606.x, PMID: 11556941 PMC1495268

[21] Kroenke K Strine TW Spitzer RL Williams JBW Berry JT Mokdad AH. The PHQ-8 as a measure of current depression in the general population. J Affect Disord. (2009) 114:163–73. doi: 10.1016/j.jad.2008.06.026, PMID: 18752852

[22] Cheng ZC Fu FW Lian YZ Zhan ZX Zhang WY. Low-carbohydrate-diet score, dietary macronutrient intake, and depression among adults in the United States. J Affect Disord. (2024) 352:125–32. doi: 10.1016/j.jad.2024.02.054, PMID: 38367707

[23] Durrleman S Simon R. Flexible regression-models with cubic-splines. Stat Med. (1989) 8:551–61. doi: 10.1002/sim.4780080504 2657958

[24] Klonsky ED May AM Saffer BY. Suicide, suicide attempts, and suicidal ideation In: Cannon TD Widiger T, editors. Annual review of clinical psychology, vol. 12 (2016). 307–30. doi: 10.1146/annurev-clinpsy-021815-093204 26772209

[25] Zhang H Li YF Torres ME. How does a suicide attempter eat differently from others? Comparison of macronutrient intakes. Nutrition. (2005) 21:711–7. doi: 10.1016/j.nut.2004.11.009, PMID: 15925296

[26] Pretorius RA Palmer DJ. High-Fiber diet during pregnancy characterized by more fruit and vegetable consumption. Nutrients. (2021) 13:35. doi: 10.3390/nu13010035 PMC7824257 33374192

[27] Hwang IC Choi S. Association between consumption of fruits and vegetables with suicidal ideation. Public Health Nutr. (2022) 25:1285–90. doi: 10.1017/S1368980021004687, PMID: 34839839 PMC9991738

[28] Li YF Zhang H McKeown RE. Cross-sectional assessment of diet quality in individuals with a lifetime history of attempted suicide. Psychiatry Res. (2009) 165:111–9. doi: 10.1016/j.psychres.2007.09.004, PMID: 19046606

[29] Kim H Ryu S Jeon HJ Roh S. Lifestyle factors and suicide risk: a nationwide population-based study. J Affect Disord. (2023) 328:215–21. doi: 10.1016/j.jad.2023.02.044, PMID: 36806600

[30] Fu JX Zheng Y Gao Y Xu WH. Dietary Fiber intake and gut microbiota in human health. Microorganisms. (2022) 10:2507. doi: 10.3390/microorganisms10122507, PMID: 36557760 PMC9787832

[31] Zhuo Y Cao M Gong YC Tang LC Jiang XM Li Y. Gut microbial metabolism of dietary fibre protects against high energy feeding induced ovarian follicular atresia in a pig model. Br J Nutr. (2021) 125:38–49. doi: 10.1017/S0007114520002378, PMID: 32600501

[32] Sivaramakrishnan S Venkatesan V Paranthaman SK Sathianathan R Raghavan S Pradhan P. Impact of serotonin pathway gene polymorphisms and serotonin levels in suicidal behavior. Med Princ Pract. (2023) 32:250–9. doi: 10.1159/000534069, PMID: 37717578 PMC10659705

[33] Encarnação JC Pires AS Amaral RA Gonçalves TJ Laranjo M Casalta-Lopes JE. Butyrate, a dietary fiber derivative that improves irinotecan effect in colon cancer cells. J Nutr Biochem. (2018) 56:183–92. doi: 10.1016/j.jnutbio.2018.02.018, PMID: 29587241

[34] Schroeder M Krebs MO Bleich S Frieling H. Epigenetics and depression: current challenges and new therapeutic options. Curr Opin Psychiatry. (2010) 23:588–92. doi: 10.1097/YCO.0b013e32833d16c1, PMID: 20644477

[35] Park RJ Kim YH. Association between high sensitivity CRP and suicidal ideation in the Korean general population. Eur Neuropsychopharmacol. (2017) 27:885–91. doi: 10.1016/j.euroneuro.2017.06.010, PMID: 28663123

[36] Sun SN Wilson CM Alter S Ge YC Hazlett EA Goodman M. Association of interleukin-6 with suicidal ideation in veterans: a longitudinal perspective. Front Psychiatry. (2023) 14:1231031. doi: 10.3389/fpsyt.2023.1231031, PMID: 37779624 PMC10540304

[37] Saghafian F Hajishafiee M Rouhani P Saneei P. Dietary fiber intake, depression, and anxiety: a systematic review and meta-analysis of epidemiologic studies. Nutr Neurosci. (2023) 26:108–26. doi: 10.1080/1028415X.2021.2020403, PMID: 36692989

